# Effects of Vitamin D Deficiency on Proliferation and Autophagy of Ovarian and Liver Tissues in a Rat Model of Polycystic Ovary Syndrome

**DOI:** 10.3390/biom9090471

**Published:** 2019-09-10

**Authors:** Krisztina Lajtai, Csilla Terézia Nagy, Róbert Tarszabó, Rita Benkő, Leila Hadjadj, Réka Eszter Sziva, Dóra Gerszi, Bálint Bányai, Péter Ferdinandy, György László Nádasy, Zoltán Giricz, Eszter Mária Horváth, Szabolcs Várbíró

**Affiliations:** 1 Department of Obstetrics and Gynecology, Semmelweis University, Üllői út 78/a, 1082 Budapest, Hungary; 2 Department of Pharmacology and Pharmacotherapy, Semmelweis University, Nagyvárad tér 4, 1089 Budapest, Hungary; 3 Department of Physiology, Semmelweis University, Tűzoltó utca 37-47, 1094 Budapest, Hungary; 4 Institute of Clinical Experimental Research, Semmelweis University, Tűzoltó utca 37-47, 1094 Budapest, Hungary

**Keywords:** hyperandrogenism, vitamin D, polycystic ovary syndrome (PCOS), insulin resistance, autophagy, oxidative stress

## Abstract

Aim: We aimed to examine the alterations of the insulin signaling pathway, autophagy, nitrative stress and the effect of vitamin D supplementation in the liver and ovaries of vitamin D deficient hyperandrogenic rats. Methods: Female Wistar rats received eight weeks of transdermal testosterone treatment and lived on a low vitamin D diet (D–T+). Vitamin D supplementation was achieved by oral administration of vitamin D3 (D+T+). Sham-treated (D+T–) and vitamin D deficient animals (D–T–) served as controls. (N = 10–12 per group). Results: D–T+ animals showed decreased LC3 II levels in the liver and increased p-Akt/Akt and p-eNOS/eNOS ratios with decreased insulin receptor staining in the ovaries. Vitamin D supplementation prevented the increase of Akt phosphorylation in the ovaries. Vitamin D deficiency itself also led to decreased LC3 II levels in the liver and decreased insulin receptor staining in the ovaries. D–T+ group showed no increase in nitrotyrosine staining; however, the ovaries of D–T– rats and the liver of D+T+ animals showed increased staining intensity. Conclusion: Vitamin D deficiency itself might lead to disrupted ovarian maturation and autophagy malfunction in the liver. Preventing Akt phosphorylation may contribute to the beneficial effect of vitamin D treatment on ovarian function in hyperandrogenism.

## 1. Introduction

Polycystic ovary syndrome (PCOS) is a complex endocrine disease of fertile women, its estimated occurrence is between 5–15%. PCOS involves hyperandrogenism and ovulatory dysfunction leading to infertility or an increased rate of miscarriage. Furthermore, it is associated with a variety of other comorbidities including obesity, type 2 diabetes mellitus (T2DM), and non-alcoholic fatty liver disease (NAFLD) [1].

Besides the characteristic hyperandrogenism, vitamin D deficiency is shown to commonly be associated with PCOS [2,3]. Both vitamin D deficiency [4] and hyperandrogenism may contribute to the development of insulin resistance (IR) observed in women with PCOS, although the underlying mechanism of IR seems to be different [5]. Vitamin D deficiency also plays role in the ovarian dysfunction of these patients [6]. The beneficial effect of vitamin D supplementation in PCOS has been previously shown on insulin resistance, lipid metabolism and accumulation as well as on ovarian function, however the results of randomized controlled trials are controversial and the underlying molecular mechanisms are not fully understood [6,7,8,9].

Alteration in oxidative stress and cell death pathways are proposed to play important role in the development of PCOS [10]. Recent studies also stipulate that inadequate regulation of autophagy in ovarian and hepatic tissues may also contribute to the pathogenesis of the disease and the related insulin resistance [11]. Inhibition of autophagy pathways as potential underlying mechanisms in skeletal muscle IR was described in an animal model of PCOS [12]. Another study reported the attenuating effect of vitamin D on cardiac abnormalities in a PCOS model by normalizing autophagy function in cardiomyocytes [13]. However, it has not been thoroughly investigated how vitamin D deficiency may alter hepatic and ovarian autophagy in PCOS.

In the present study, we aimed to examine the effect of vitamin D supplementation on insulin receptor signaling, autophagosomal turnover and nitrative stress in the liver and the ovaries in a vitamin D deficient hyperandrogenic rat model of PCOS.

## 2. Materials and Methods

### 2.1. Animals

The investigation conforms to the *Guide for the Care and Use of Laboratory Animals* published by the US National Institutes of Health (8th edition, 2011) and the EU conform Hungarian Law on Animal Care (XXVIII/1998). The institutional Animal Care Commission approved the study protocol (IRB: 8/2014 PEI/001/1548-3/2014).

A total number of 46 female Wistar rats (Charles River Ltd., AnimaLab, Vác, Hungary) aged 21–28 days, weighing 100–140 g were divided into four groups as the following: Hyperandrogenic vitamin D deficient group (D–T+, n = 11); hyperandrogenic vitamin D supplemented group (D+T+, n = 12); vitamin D deficient group (D–T–, n = 11) and vitamin D supplemented group (D+T–, n = 12).

To induce hyperandrogenism, an 8-week-long transdermal testosterone treatment was implemented by applying 0.0333 mg/g of Androgel (50 mg/5 mL gel by Lab. Besins International S.A., Monaco) 5 times a week on a previously shaved 3 × 3 cm area on the back of the animals. Transdermal testosterone treatment was calculated to reach target androgen levels of previous PCOS models considering the bioefficacy of the transdermal application [14,15,16,17,18].

**Figure 1:**
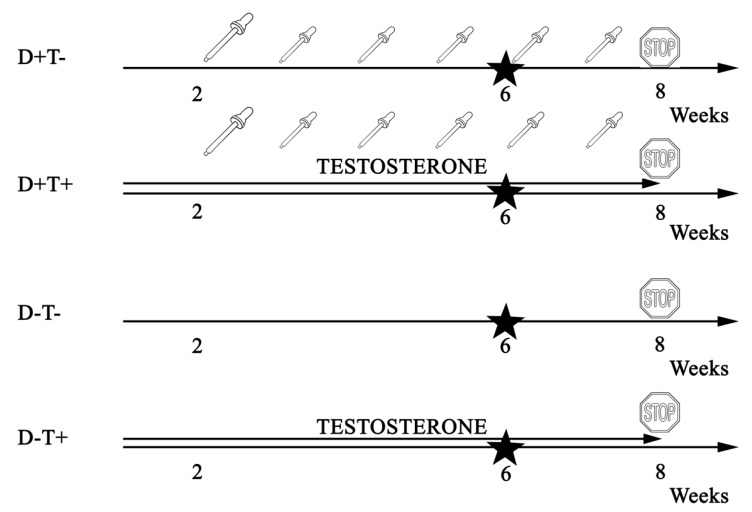
Timeline of chronic treatment in different treatment groups. Adolescent Wistar rats were divided into four groups—half of the animals received transdermal testosterone treatment (0.0333 mg/body weight grams 5 times weekly) for 8 weeks, while another half did not receive androgens. Half of the animals in each group were fed low vitamin D chow while the other half received adequate vitamin D supplementation (weekly 1.4 NE/body weight grams per os on the 3rd, 4th, 5th, 6th, and 7th week after a 500 NE saturation on the 2nd week). On the 6th treatment week, an oral glucose tolerance test (OGTT) was performed. On the 8th treatment week, the animals were sacrificed, and their tissues were kept at −80 °C until further studies.

Vitamin D depleted animals were fed the Vitamin D Free Lab Rat/Mouse Chow (ssniff Spezialdiaten GmbH, Soest, Germany) containing less than 5 IU/kg of vitamin D3, whereas supplemented animals received a regular chow containing 1000 IU/kg of vitamin D. The supplemented animals also received additional oral vitamin D (Vigantol (cholecalciferol) 20,000 IU/mL, Merck/Merck Serono, Darmstadt, Germany) as the following: 500 IU on the second week of treatment as a saturation dose, and weekly 140 IU/100 g on the fifth, sixth and seventh week of the treatment. The previously calculated amount of Vigantol was measured with micropipettes and administered to the animals using a gavage cannula. Target serum 25-OH-cholecalciferol levels (20–50 ng/mL) were adjusted to the currently accepted human vitamin D supplementation guidelines [19,20]. Figure 1 summarizes the chronic treatment of different groups week by week.

The animals were supplied with appropriate rat chow and tap water ad libitum. Rats were housed at constant room temperature (22 ± 1 °C) in 12 h/12 h light–dark cycle.

On the 8th treatment week, animals were sacrificed in Nembutal anaesthesia (45 mg/kg intraperitoneal). Testosterone-treated animals had no estrus cycles; D+T– and D–T– animals were selected regardless of estrus cycle phase. Liver and ovarian tissue samples were harvested after whole-body perfusion, snap frozen in liquid nitrogen and stored at −80 °C or fixed in formaldehyde (4%) phosphate buffered saline solution. Fixed tissues were embedded in paraffin and histological sections (5 µm) were prepared.

### 2.2. Immunohistochemistry

Immunohistochemistry was performed on paraffin embedded tissue sections of the liver and ovaries against insulin receptor beta (IRβ), vitamin D receptor (VDR) and 3-nitrotyrosine (NT) by BenchMark ULTRA Automated IHC/ISH slide staining system (Ventana Medical Systems, Inc., Tucson, AZ, USA) using monoclonal mouse anti-IRβ (Santa Cruz Biotechnology, Dallas, TX, USA), polyclonal rabbit anti-VDR and anti-NT (Abcam, Cambridge, UK) antibodies. Visualization of specific labeling with diaminobenzidine (DAB) as colored substrate and hematoxylin counterstaining was achieved by UltraView Universal DAB Detection Kit (Ventana Medical Systems, Inc.). Microscopic images of immunostained tissue sections were taken by Zeiss Axio Imager system (Zeiss, Oberkochen, Germany). Non-calibrated optical density of specific staining was measured by ImageJ software (NIH, Bethesda, MA, USA).

### 2.3. Western Blotting

Tissue samples were homogenized in radioimmunoprecipitation assay buffer (Cell Signaling, Danvers, MA, USA) supplemented with protease inhibitor (Roche, Basel, Switzerland) and sodium fluoride (Sigma, St Louis, MO, USA). Protein concentration of the homogenates was measured by BCA kit (Thermo Scientific, Waltham, MA, USA). An equal amount of protein (40 µg) was mixed with Pierce™ Lane Marker Reducing Sample Buffer (Thermo Scientific), loaded and separated in a 4–20% precast Tris-glycine SDS polyacrilamide gel (Bio-Rad, Hercules, CA, USA). Proteins were transferred onto a polyvinylidene difluoride membrane (Bio-Rad) at 200 mA, overnight. Proper transfer was visualized with Ponceau staining (Sigma). Membranes were blocked with 5% bovine serum albumin (BSA; Santa Cruz Technology) or with 5% Blotting-Grade Blocker in Tris-buffered saline containing 0.05% Tween-20 (0.05% TBS-T; Sigma) at room temperature for 2 h. Membranes were probed with primary antibodies purchased from Cell Signaling overnight at 4 °C (LC3A/B—#4108; phospho-S6 [Ser235/236]—#2211; S6—#2317; phospho-Akt [Ser473]—#9271; Akt—#9272; phospho-eNOS—#9571; eNOS—#9572; GAPDH—#5174), and with corresponding HRP-conjugated secondary antibodies (Cell Signaling) for 2 h at room temperature. Signals were detected with an enhanced chemiluminescence kit (Bio-Rad) by Chemidoc XRS+ (Bio-Rad). Antibodies detecting phosphorylated epitopes were removed with Pierce Stripping Buffer (Thermo Scientific) before incubation with antibodies detecting the total protein.

### 2.4. Statistical Analysis

Effects of testosterone treatment and vitamin D status were evaluated by two-way ANOVA using Tukey’s post-hoc test by Prism 5 (GraphPad, GraphPad Software, San Diego, CA, USA). In case of significant interaction between the effects of the two treatments (NT staining), one-way ANOVA with Tukey’s post hoc test was performed. *p* < 0.05 was uniformly accepted as the threshold for statistical significance. Data are shown as mean ± SEM.

## 3. Results

### 3.1. Chracteristics of PCOS in Our Model

The physiological parameters describing our model are discussed in detail in Hadjadj L et al. (2018) [5]. Briefly, testosterone treatment resulted in a 10-fold increase in testosterone and a 5-fold increase in DHT serum levels while non-treated animals had normal androgen levels. Vitamin D supplementation resulted in adequate serum 25-OH-vitamin D levels (target range: 20–50 ng/mL) whereas animals kept on a vitamin D free diet showed severe vitamin D deficiency (serum levels under 10 ng/mL) [19]. Testosterone treatment led to an elevated number of smaller, mostly primordial follicles that is typical for PCOS, and reduced serum progesterone levels caused by missing luteinisation. We detected an emerging insulin resistance with testosterone treatment elevating glucose levels and vitamin D deficiency elevating insulin levels and leading to a higher computed HOMA-IR (Homeostatic Model of Insulin Resistance) value following OGTT.

### 3.2. Alterations of Insulin Receptor Signaling and Autophagy in the Liver

**Figure 2:**
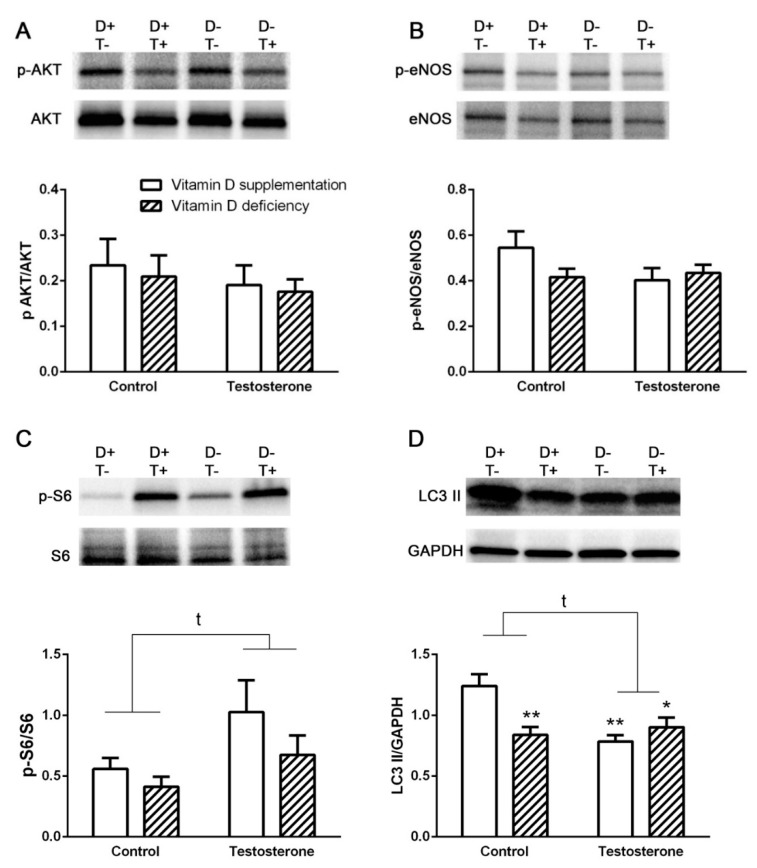
Alterations in insulin signaling pathway and autophagy in the liver. (**A**) Akt phosphorylation in liver tissue. (**B**) eNOS phosphorylation in liver tissue. (**C**) Protein S6 phosphorylation in the liver. (**D**) LC3 II formation in the liver. LC3 II levels normalized to GAPDH. Data are presented as mean ± SEM. Two-way (factor 1—vitamin D supplementation vs. vitamin D deficiency; factor 2—control vs. testosterone treatment) ANOVA; t: *p* < 0.05 control vs. testosterone treatment; Tukey’s post hoc test (comparison of experimental groups), * *p* < 0.05 vs. D+T– group, ** *p* < 0.01 vs. D+T– group; n = 5–8 in each group.

Vitamin D deficient hyperandrogenic animals showed reduced LC3 II levels reflecting decreased autophagosomal turnover. Vitamin D supplementation failed to ameliorate this change. However, vitamin D deficiency itself also led to decreased LC3 II levels (Figure 2D).

Akt phosphorylation was not altered in any of the diseased groups (Figure 2A). Besides Akt phosphorylation eNOS phosphorylation was also unaltered by testosterone treatment or vitamin D deficiency, suggesting unchanged insulin signaling (Figure 2A,B).

On the other hand, S6 protein phosphorylation was increased by testosterone treatment that may reflect a direct proliferative effect of testosterone (Figure 2C).

### 3.3. Alterations of Insulin Receptor Signaling and Autophagy in the Ovaries

**Figure 3:**
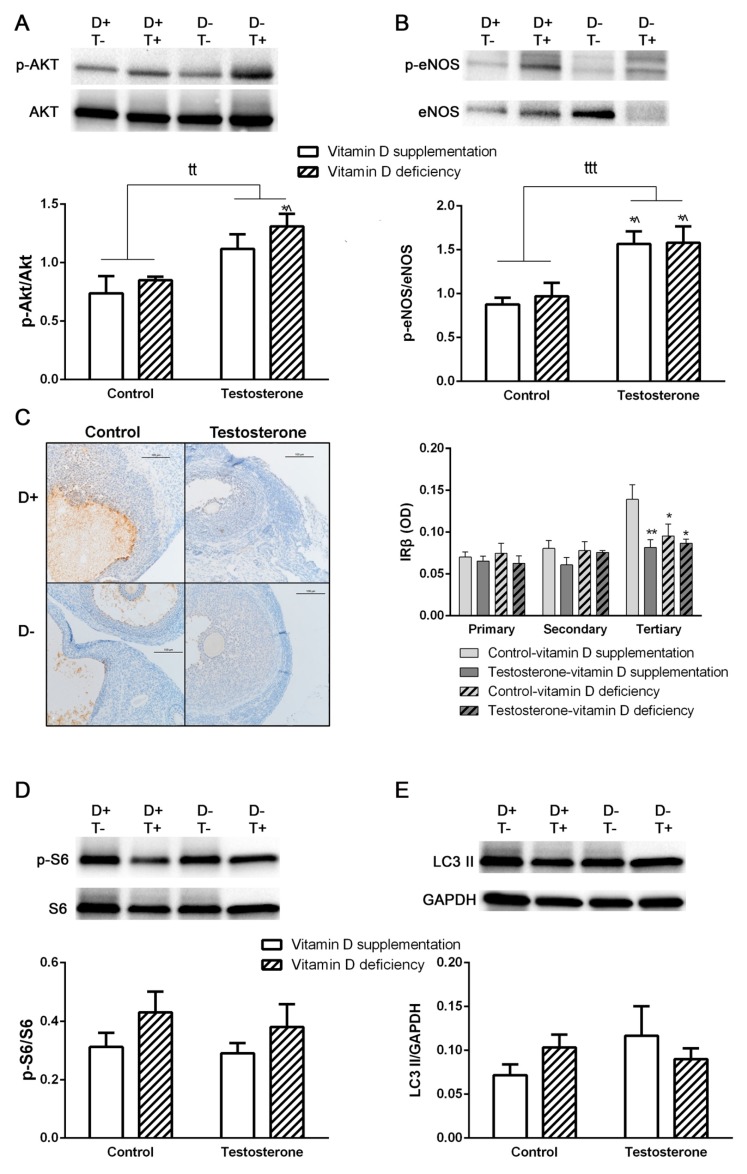
Alterations in insulin signaling pathway and autophagy in ovarian tissue. (**A**) Akt phosphorylation in ovarian tissue. (**B**) eNOS phosphorylation in ovarian tissue. (**C**) Immunohistochemical staining of ovarian tissue with anti-IRβ antibody. (**D**) Protein S6 phosphorylation in the ovary. (**E**) LC3 II formation in ovarian tissue. For Panel A–B and D–E: Data are presented as mean ± SEM. Two-way (factor 1—vitamin D supplementation vs. vitamin D deficiency, factor 2—control vs. testosterone treatment) ANOVA, tt: *p* < 0.01 control vs. testosterone treatment, ttt: *p* < 0,001 control vs. testosterone treatment; Tukey’s post hoc test (comparison of experimental groups) * *p* < 0.05 vs. D+T– group, ^ *p* < 0.01 vs. D–T– group; n = 5–8 in each group. For Panel D: Data are presented as mean ± SEM. Two-way (factor 1—follicle size, factor 2—experimental group) ANOVA; Tukey’s post hoc test (comparison of experimental groups) * *p* < 0.05 vs. D+T– group among large follicles, ** *p* < 0.01 vs. D+T– group among large follicles; n = 5–8 in each group.

We detected a significantly increased p-Akt/Akt and p-eNOS/eNOS ratio in the ovarian samples of vitamin D deficient hyperandrogenic animals. Vitamin D supplementation prevented the elevation of p-Akt/Akt ratio, but not the p-eNOS/eNOS ratio. Vitamin D deficiency itself had no effect on these parameters (Figure 3A,B). Having examined the total area of the ovaries, we found no difference between study groups in insulin receptor beta (IRβ) immunostaining. Literature suggests difference in IRβ staining of follicles when in various stages of maturation [21]. According to our results, in the primordial, primary and secondary follicles, there was no difference in IRβ positivity. On the other hand, among the large antral follicles, there was a significantly decreased IRβ immunostaining in all experimental groups (D–T–, D+T+, D–T+) compared to sham treated controls (D+T–) (Figure 3C). The ratio of p-S6/S6 and the level of LC3 II was not influenced by either treatment (Figure 3D,E).

### 3.4. Nitrative Stress in the Liver and in the Ovaries

**Figure 4:**
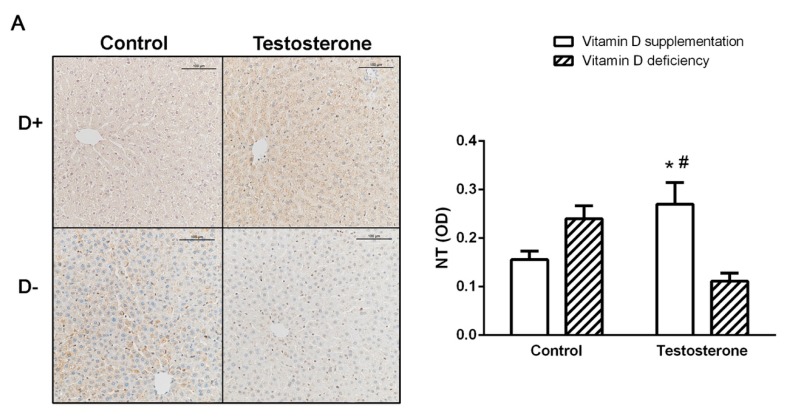

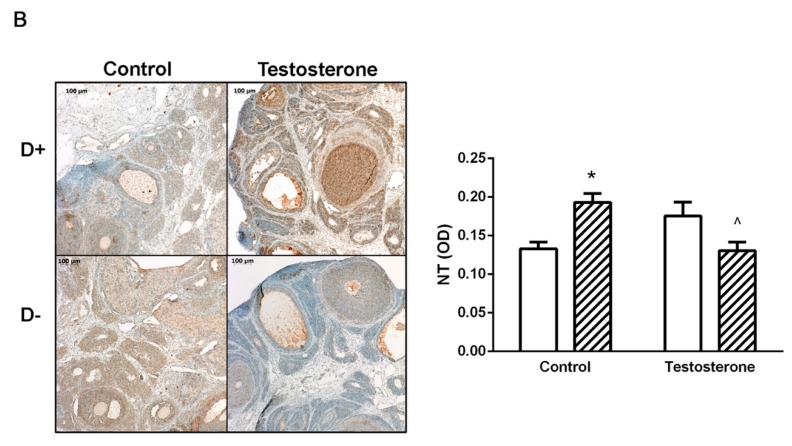
Nitrative stress in the liver and in the ovaries. (**A**) Immunohistochemical staining of liver tissue with anti-NT antibody. (**B**) Immunohistochemical staining of ovarian tissue with anti-NT antibody. Data are presented as mean ± SEM. One-way ANOVA with Tukey’s post hoc test. * *p* < 0.05 vs. D+T– group, # *p* < 0.05 vs. D–T+ group, ^ *p* < 0.05 vs. D–T– group; n = 5–8 in each group.

We found significantly increased density of 3-nitrotyrosine (NT) staining in the liver tissue of the vitamin D supplemented hyperandrogenic (D+T+) group. However, in the ovaries, increased staining was observed in the vitamin D deficient group (D–T–). On the other hand, the vitamin D deficient hyperandrogenic rats (D–T+) did not show increased nitrative stress in the examined tissues (Figure 4A,B).

### 3.5. Changes of Vitamin D Receptor Immunostaining in the Liver and the Ovaries

**Figure 5:**
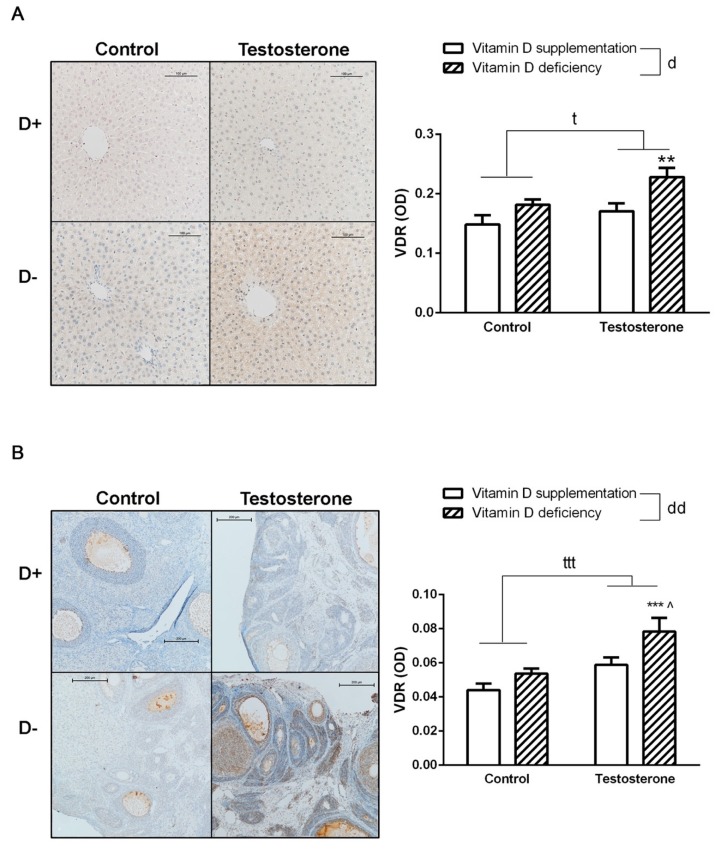
Vitamin D receptor density in the liver and in the ovaries. (**A**) Immunohistochemical staining of liver tissue with anti-VDR antibody. (**B**) Immunohistochemical staining of ovarian tissue with anti-VDR antibody. Data are presented as mean ± SEM. Two-way (factor 1—vitamin D supplementation vs. vitamin D deficiency, factor 2—control vs. testosterone treatment) ANOVA, t: *p* < 0.05 control vs. testosterone treatment, ttt: *p* < 0,001 control vs. testosterone treatment, d: *p* < 0.05 vitamin D supplementation vs. vitamin D deficiency, dd: *p* < 0.01 vitamin D deficiency vs. vitamin D deficiency; Tukey’s post hoc test (comparison of experimental groups), ** *p* < 0.01 vs. D+T– group,*** *p* < 0.001 vs. D+T– group, ^ *p* < 0.05 vs. D–T– group; n = 5–8 in each group.

In vitamin D deficient hyperandrogenic animals, increased VDR specific staining intensity was observed in both hepatic and ovarian tissues. Vitamin D supplementation prevented this change (Figure 5A,B).

## 4. Discussion

The risk of NAFLD that is strongly linked to metabolic syndrome is known to be increased in PCOS due to elevated androgen levels and insulin resistance [22]. The role of autophagy in the pathogenesis of NAFLD has been extensively studied recently. Decreased autophagosomal turnover is shown to play important role in the impairment of lipid droplet degradation in the liver [23]. In our vitamin D deficient hyperandrogenic rat model, we found decreased LC3II levels indicating reduced autophagosomal turnover in the liver. This may suggest the involvement of decreased autophagy in the increased risk of NAFLD in PCOS.

Increased Akt/mTOR signaling may contribute to the progression of steatosis in case of higher insulin levels in insulin resistance by inhibiting autophagy [23]. On the other hand, in our vitamin D deficient hyperandrogenic rat model, the decreased autophagosomal turnover characterized by decreased LC3II levels was not accompanied by increased Akt phosphorylation. Therefore, the decreased autophagosomal turnover was probably not due to increased Akt signaling.

Vitamin D was previously shown to attenuate hepatic steatosis by inducing autophagy [24]. However, in our experiments, vitamin D supplementation failed to prevent the reduction in LC3II levels during testosterone treatment. On the other hand, vitamin D deficiency itself without hyperandrogenism resulted in reduced LC3II levels. The decreased LC3II levels was not accompanied with increased AKT phosphorylation in this case either, suggesting an Akt independent mechanism of vitamin D action. One possible mechanism is the upregulation of autophagy-related 16-like 1 (ATG16L1) gene that was shown to play role in the autophagy inducing effect of vitamin D in mice fed with high fat diet [24]. The characterization of mechanisms responsible for decreased hepatic autophagy in hyperandrogenism and vitamin D deficiency, however, requires further investigations.

Besides Akt phosphorylation eNOS phosphorylation was also unaltered by testosterone treatment or vitamin D deficiency, suggesting unchanged insulin signaling. Vitamin D deficient animals of the present rat model are characterized by elevated insulin levels [5] where unaltered Akt and eNOS phosphorylation may reflect insulin resistance in the liver.

At the same time, in our experiments, S6 protein phosphorylation was increased by testosterone treatment. This may reflect a direct proliferative effect of testosterone that can also contribute to the development of NAFLD independently from insulin signaling pathway [25]. It has also been previously shown that liver specific depletion of the p70 S6 kinase protect against hepatic steatosis, suggesting the role of S6 phosphorylation in the development of NAFLD. The activation of the mTOR/S6 pathway in the liver may also contribute to the development of systemic insulin resistance [26].

In contrast to the liver, elevated Akt and eNOS phosphorylation was found in the ovarian tissues of vitamin D deficient hyperandrogenic rats. Low-dose dihydrotestosterone treatment in mice also led to similar changes in Atk phosphorylation; p-Akt/Akt ratio was increased in the ovaries, but not in the liver [27]. Altered Akt activation disturbs dominant follicle selection, as by the time of selection, dominant follicle normally have significantly elevated p-Akt/Akt ratio compared to subordinate follicles [28]. The increased activity of PI3K/Akt pathway may also contribute to the development of polycystic ovaries, as the overactivation of this pathway can lead do impaired follicle development and the appearance of numerous immature follicles and may contribute to cyst formation [29]. Vitamin D supplementation managed to prevent the increase in Akt phosphorylation that may play a role in the beneficial effect of vitamin D supplementation for follicular function.

On the other hand, eNOS phosphorylation was not prevented by vitamin D supplementation in hyperandrogenic rats. Whereas the acute activation of eNOS by testosterone through the phosphatidylinositol 3 kinase (PI3K)/Akt pathway is well characterized [30], the chronic effect of androgens on eNOS phospholytaion is rarely examined [31]. In penile tissues, increased eNOS activity was found after elevation of serum testosterone levels by chronic treatment of icariin [32].

IRβ can be found on granulosa and theca cells in the ovaries [33] and its expression changes during folliculogenesis; upregulation of IRβ can be observed during maturation and gets prominent in tertiary antral follicles [34]. This characteristic upregulation was not present in hyperandrogenic vitamin D deficient rats, however, vitamin D supplementation failed to prevent this effect of testosterone treatment. Vitamin D deficiency itself resulted in significant reduction of IRβ immunostaining of tertiary follicles. Disturbance in the IRβ expression in PCOS [21] has been associated with receptor downregulation due to hyperinsulinaemia. Supporting this theory, in our study, the vitamin D-deficient rats had elevated insulin levels compared to vitamin D-supplemented groups. However, D+T+ animals had higher plasma glucose levels without hyperinsulinemia, they also showed lower IRβ density in large follicles. The increased Akt phosphorylation found in testosterone treated rats may play role in this phenomenon through the phosphorylation of the adapter protein APS inducing ubiquitination, and consequent internalization and degradation of IRβ [35,36]. Although the hyperinsulinemia in vitamin D deficiency and the possible increase in IRβ degradation induced by the elevated p-Akt/Akt ratio in testosterone treatment may play role in lower IRβ density, further investigations are necessary to clarify the involved mechanisms.

PCOS is associated with enhanced inflammatory activity and systemic oxidative and nitrative stress [37]. In our experiment, Vitamin D deficiency alone increased NT staining in the ovaries, whereas in the liver testosterone treatment had a similar effect. The role of mTOR and p-S6 pathway has been previously shown to be involved in inflammatory processes leading to iNOS induction and oxidative-nitrative stress [38]. S6 phosphorylation showed similar pattern as NT staining in our experiments, although its changes did not reach significant level in all cases. Another experiment also found significant NT increase in brain homogenates of vitamin D-deficient mice [39]. Surprisingly, in our setting, D–T+ group showed no increase in NT staining. This could be explained by exhaustion of nitrosative stress, furthermore in PCOS women, a significantly decreased serum level of arginine and citrulline was measured, suggesting a lack of substrate for iNOS [40] and the consequential protein nitration.

In our experiment, elevated expression of VDR is detected in both ovarian and liver tissues of vitamin D deficient hyperandrogenic rats, which was prevented by vitamin D supplementation. In spite of the generally accepted idea that vitamin D upregulates VDR expression [41], in recently published experiments, an elevated VDR expression was detected in the heart tissue of vitamin D deficient rats [42]. Moreover, decreased VDR mRNA levels were detected in the peripheral blood mononuclear cells of vitamin D supplemented multiple sclerosis patients [43]. The effect of testosterone on VDR expression was examined in a recent study, but they found no difference in the number of VDR in porcine ovarian follicular cells after a 6 h testosterone treatment. However, disrupted coupling with retinoid X receptor (RXR) was detected, causing an alteration in the transcriptional activity of VDR [44]. Testosterone was also shown to increase the expression of vitamin D binding protein in male rats [45]. Research involving prostate and ovarian cancer propose the potential role of androgen receptor-coregulators, especially ARA70 (androgen receptor-associated protein 70), which seems to be necessary for both androgen receptor (AR) and VDR transcriptional activity. High testosterone levels lead to elevated AR activation, limiting the availability of ARA70 to coregulate the VDR/RXR complex [46]. VDR/RXR complex stimulates sulfotransferase family 2A member 1 (SULT2A1) and cytochrome P450 3A4 (CYP3A4) transcription, both of which are responsible for deactivating free testosterone [47,48]. This way testosterone treatment may lead to the worsening of its own clearance inducing a vicious circle of hyperandrogenism.

## 5. Conclusions

**Figure 6:**
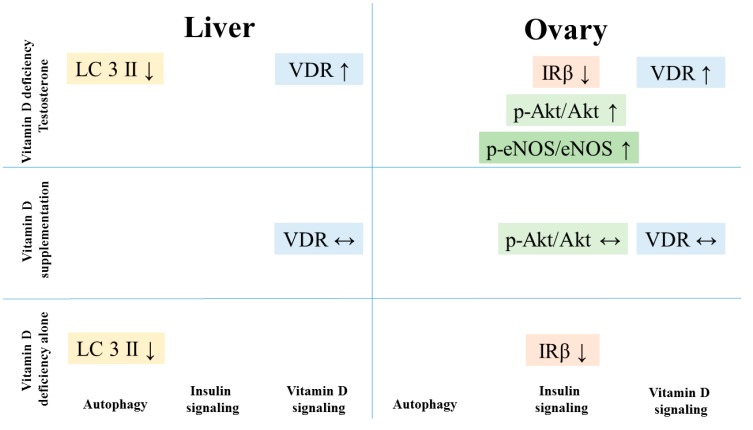
Summary of the changes in receptor density, insulin signaling pathway and autophagy in different treatment groups.

Altered insulin signaling and autophagosomal turnover in connection with nitrative stress was found in the liver and ovarian tissues of our rat model of PCOS (Figure 6). Combined hyperandrogenism and vitamin D deficiency led to decreased autophagy in the liver and altered insulin signaling in the ovaries. Vitamin D supplementation could prevent the increase in Akt phosphorylation in the ovaries that may contribute to the beneficial effect of vitamin D treatment on ovarian function. However, it could not reverse the other adverse effects observed in the double challenged group. On the other hand, vitamin D deficiency itself led to disrupted ovarian maturation and autophagy malfunction in the liver. Autophagy might be a key component in cellular changes induced by hyperandrogenism or vitamin D deficiency that plays role in the development of NAFLD in PCOS patients.
